# Suppressing STAT3 activity protects the endothelial barrier from VEGF-mediated vascular permeability

**DOI:** 10.1242/dmm.049029

**Published:** 2021-11-11

**Authors:** Li Wang, Matteo Astone, Sk. Kayum Alam, Zhu Zhu, Wuhong Pei, David A. Frank, Shawn M. Burgess, Luke H. Hoeppner

**Affiliations:** 1 The Hormel Institute, University of Minnesota, Austin, MN 55912, USA; 2 Translational and Functional Genomics Branch, National Human Genome Research Institute, National Institutes of Health, Bethesda, MD 20814, USA; 3 Department of Medical Oncology, Dana-Farber Cancer Institute, Boston, MA 02215, USA; 4 Masonic Cancer Center, University of Minnesota, Minneapolis, MN 55455, USA

**Keywords:** Vascular permeability, VEGF, STAT3, Pyrimethamine, Zebrafish, COVID-19

## Abstract

Vascular permeability triggered by inflammation or ischemia promotes edema, exacerbates disease progression and impairs tissue recovery. Vascular endothelial growth factor (VEGF) is a potent inducer of vascular permeability. VEGF plays an integral role in regulating vascular barrier function physiologically and in pathologies, including cancer, stroke, cardiovascular disease, retinal conditions and COVID-19-associated pulmonary edema, sepsis and acute lung injury. Understanding temporal molecular regulation of VEGF-induced vascular permeability will facilitate developing therapeutics to inhibit vascular permeability, while preserving tissue-restorative angiogenesis. Here, we demonstrate that VEGF signals through signal transducer and activator of transcription 3 (STAT3) to promote vascular permeability. We show that genetic STAT3 ablation reduces vascular permeability in STAT3-deficient endothelium of mice and VEGF-inducible zebrafish crossed with CRISPR/Cas9-generated Stat3 knockout zebrafish. Intercellular adhesion molecule 1 (ICAM-1) expression is transcriptionally regulated by STAT3, and VEGF-dependent STAT3 activation is regulated by JAK2. Pyrimethamine, an FDA-approved antimicrobial agent that inhibits STAT3-dependent transcription, substantially reduces VEGF-induced vascular permeability in zebrafish, mouse and human endothelium. Collectively, our findings suggest that VEGF/VEGFR-2/JAK2/STAT3 signaling regulates vascular barrier integrity, and inhibition of STAT3-dependent activity reduces VEGF-induced vascular permeability.

This article has an associated First Person interview with the first author of the paper.

## Abstract (precis)

**Summary:** Genetic STAT3 ablation in mice and VEGF-inducible zebrafish reveals that VEGF signals through STAT3 to promote vascular permeability. Pyrimethamine reduces VEGF-induced permeability in animal models.

## INTRODUCTION

Proper physiological function relies on the vascular system to distribute oxygenating blood to all tissues, return deoxygenated blood to the lungs and maintain tissue homeostasis, including functions such as hemostasis, lipid transport and immune surveillance. In pathological conditions, vasculature is often adversely affected by the disease process, resulting in vascular permeability (Park-Windhol and D'Amore, 2016). Vascular endothelial growth factor (VEGF-A isoform, henceforth referred to as VEGF) is a central mediator of vascular permeability. In fact, VEGF was initially discovered as a tumor-secreted factor that strongly promotes microvascular permeability named ‘vascular permeability factor’ (Senger et al., 1983) before its subsequent identification as an endothelial mitogen essential for the development of blood vessels (Carmeliet et al., 1996; Ferrara et al., 1996; Leung et al., 1989; Nasevicius et al., 2000). In cancer, VEGF-induced vascular permeability of plasma proteins creates a matrix amenable to vascular sprouting and tumor growth (Senger et al., 1983).

In addition to driving tumor angiogenesis, VEGF stimulates tumor cell extravasation, an important step in metastasis that enables cancer cells to enter the bloodstream and potentially invade other tissues (Weis et al., 2004). Increased VEGF expression promotes hyperpermeability, edema and tissue damage leading to the pathogenesis of cardiovascular disease, cerebrovascular conditions, retinal disorders and acute lung injury. Acute lung injury, including acute respiratory distress syndrome, is among the most severe pathologies caused by coronavirus disease 2019 (COVID-19) and results in pulmonary edema caused by impaired vascular barrier function (Wu and McGoogan, 2020; Teuwen et al., 2020). Autopsy reports of deceased COVID-19 patients commonly describe severe pulmonary mucus exudation, and acute lung injury is frequently a cause of death in severe cases of COVID-19 (Gallelli et al., 2020; Xu et al., 2020). A definitive treatment for acute lung injury does not exist.

Although therapeutically inhibiting vascular permeability reduces subsequent edema and tissue damage, VEGF-mediated angiogenesis is a key tissue repair mechanism (van Bruggen et al., 1999; Navaratna et al., 2009). Therefore, temporal VEGF modulation must be achieved when administering therapies to reduce edema and repair ischemic tissue damaged by pathogenesis, which underscores the importance of fully understanding the molecular and temporal regulation of vascular permeability *in vivo*.

Signal transducer and activator of transcription (STAT) proteins regulate a wide array of cellular functions, including proliferation, differentiation, inflammation, angiogenesis and apoptosis (Huynh et al., 2019). Like most of its six other STAT protein family members, STAT3 was identified as part of a cytokine signaling cascade that potentiates the interleukin-6 (IL-6)-mediated hepatic acute phase response as a transcription factor (Akira et al., 1994; Zhong et al., 1994). In addition to its prominent role in IL-6 signal transduction, it is well established that VEGF signals primarily through VEGF receptor 2 (VEGFR-2; also known as KDR) to stimulate STAT3 activation, dimerization, nuclear translocation and DNA binding to regulate the transcription of genes involved in endothelial activation, vascular inflammation and a variety of other biological processes (Kostromina et al., 2010; Bartoli et al., 2000; Simons et al., 2016). Activation of STAT3 occurs through phosphorylation of tyrosine residue Y705 (Sasse et al., 1997). Many STAT family members are phosphorylated by Janus kinases (JAKs), which are activated through *trans*-phosphorylation following ligand-mediated receptor multimerization. Mammalian JAK family members include JAK1, JAK2, JAK3 and TYK2 (Rawlings et al., 2004). Reports across a variety of tumor and endothelial cell types have suggested members of the JAK family, SRC and the intrinsic kinase activity of VEGFR-2 as VEGF-induced activators of STAT3 (Bartoli et al., 2003; Chen et al., 2008; Yahata et al., 2003; Yeh et al., 2006; Zhao et al., 2015). However, the precise mechanism through which VEGF/VEGFR-2 signaling promotes phosphorylation of STAT3 is poorly understood and likely tissue- and cell-type specific.

Here, we identify STAT3 as a central mediator of VEGF-induced vascular permeability. In our study, we exploit the strengths of three model systems – zebrafish, mice and cultured human endothelial cells – to investigate the role of STAT3 in vascular permeability mediated by VEGF and VEGFR-2 signaling. Zebrafish (*Danio rerio*) have emerged as an invaluable vertebrate model of human pathophysiology due to their genetic similarity to *Homo sapiens* and the transparency of embryos that makes zebrafish amenable to *in vivo* fluorescent imaging, among other reasons (Astone et al., 2017). We crossed previously described transgenic heat-inducible VEGF zebrafish (Hoeppner et al., 2012, 2015) to CRISPR/Cas9-generated Stat3 genomic knockout zebrafish (Pei et al., 2018) to evaluate the role of Stat3 in VEGF-induced vascular permeability *in vivo*. Furthermore, we established a complementary VEGF-mediated vascular permeability model in endothelial cell-specific STAT3 knockout mice, demonstrate that multiple pharmacological inhibitors of STAT3 reduce vascular permeability *in vivo*, and describe the molecular regulation of VEGF-induced vascular barrier integrity in human endothelial cells.

## RESULTS

### VEGF/VEGFR-2 induces STAT3 phosphorylation and nuclear localization

Given that we sought to investigate STAT3 as an important regulator of VEGF-induced vascular permeability, we first performed studies to confirm that VEGF activates STAT3 in endothelium. We observed activation of VEGFR-2 and STAT3 following VEGF stimulation (Fig. S1A), which corresponds with prior reports (Chen et al., 2008). VEGF stimulation promotes physical interaction of VEGFR-2 and STAT3, as evident by immunoprecipitation studies demonstrating an association of total VEGFR-2 and total STAT3 (Fig. S1B) as well as VEGF-dependent interactions between the phosphorylated forms of VEGFR-2 and STAT3 (Fig. S1C). Immunofluorescence studies demonstrate that STAT3 translocates to the nucleus upon VEGF/VEGFR-2-mediated activation in human umbilical vein endothelial cells (HUVECs) (Fig. S1D,E). Collectively, our results suggest that VEGF stimulates VEGFR-2 to induce STAT3 phosphorylation and nuclear localization in human endothelial cells.

### VEGF-induced vascular permeability is reduced in Stat3-deficient zebrafish

**Fig. 1.**
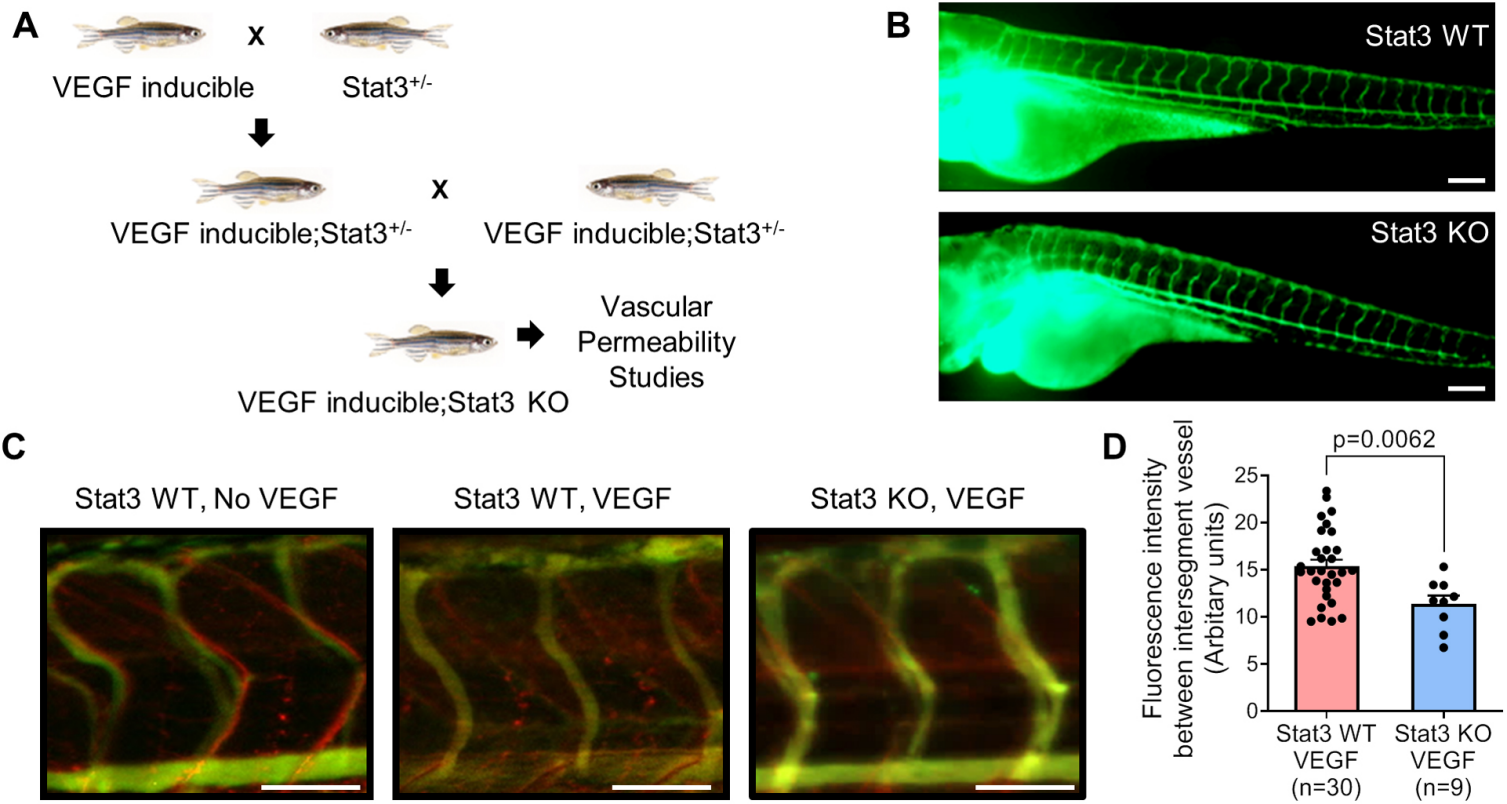
**VEGF-induced vascular permeability is reduced upon CRISPR/Cas9-mediated knockout of Stat3 in zebrafish.** (A) VEGF-inducible zebrafish were crossed to Stat3^+/−^ (heterozygous) zebrafish to generate VEGF-inducible; Stat3^+/−^ double transgenic fish, which were intercrossed to generate VEGF-inducible; Stat3^−/−^ (KO) zebrafish. (B) CRISPR/Cas9-generated Stat3 KO zebrafish (bottom) display no overt vascular defects relative to wild-type (WT) zebrafish (top). The vascular system of 3 days post-fertilization (dpf) zebrafish was visualized by microangiography with 2000 kDa FITC-dextran. Representative images of at least three zebrafish per group are shown. Scale bars: 100 μm. (C) Microangiography using 70 kDa Texas Red-dextran permeabilizing tracer (red) and 2000 kDa FITC-dextran intersegmental vessel marker (green) was performed on 3 dpf Stat3**^+/+^** (negative controls without VEGF induction; left)**,** VEGF-induced, Stat3**^+/+^** (middle) and VEGF-induced, Stat3**^−/−^** (right) zebrafish. Representative images shown were obtained using a Zeiss Apotome 2 microscope with a Fluar 5×/0.25 NA lens at room temperature (RT). Scale bars: 50 μm. (D) Quantitative analysis of vascular permeability upon VEGF stimulation in WT Stat3**^+/+^** (*n*=30) and KO Stat3**^−/−^** (*n*=9) zebrafish. Mean±s.e.m., unpaired, two-tailed Student's *t*-test.

To investigate the role of STAT3 in VEGF-induced vascular permeability *in vivo*, we utilized a heat shock-inducible VEGF transgenic zebrafish (iVEGF) that we previously developed to identify regulators of VEGF-mediated vascular leakage (Hoeppner et al., 2012, 2015). Using this iVEGF zebrafish model of vascular permeability, we previously established that heat induction of the *VEGF* transgene results in significant fluorescent dextran leakage from intersegmental vessels into the extravascular space (Hoeppner et al., 2012, 2015). Here, we generated Stat3 knockout zebrafish with an inducible *VEGF* transgene by crossing iVEGF zebrafish (Hoeppner et al., 2012) to CRISPR/Cas9-generated Stat3 genomic knockout (Stat3 KO) zebrafish (Pei et al., 2018) (Fig. 1A; Fig. S2). Importantly, we did not observe any vascular development defects in Stat3 KO larval zebrafish, and the vascular system of Stat3 KO zebrafish at 3 days post-fertilization (dpf) is indistinguishable from that of wild-type Stat3^+/+^ siblings (Fig. 1B). To assess vascular permeability, VEGF was heat induced in Stat3 KO; iVEGF zebrafish following ventricular co-injection of 70 kDa Texas Red-dextran as a permeabilizing tracer and 2000 kDa FITC-dextran as a marker of the veins. Zebrafish were immediately live imaged to measure vascular permeability, evident by leakage of Texas Red-dextran into the extravascular space (Hoeppner et al., 2012). Using these techniques, our data showed decreased VEGF-induced vascular permeability in Stat3 KO zebrafish relative to corresponding wild-type controls (Fig. 1C,D), suggesting that VEGF signals through STAT3 to promote vascular permeability.

### Endothelial-specific STAT3 knockout mice exhibit decreased VEGF-induced vascular permeability

**Fig. 2.**
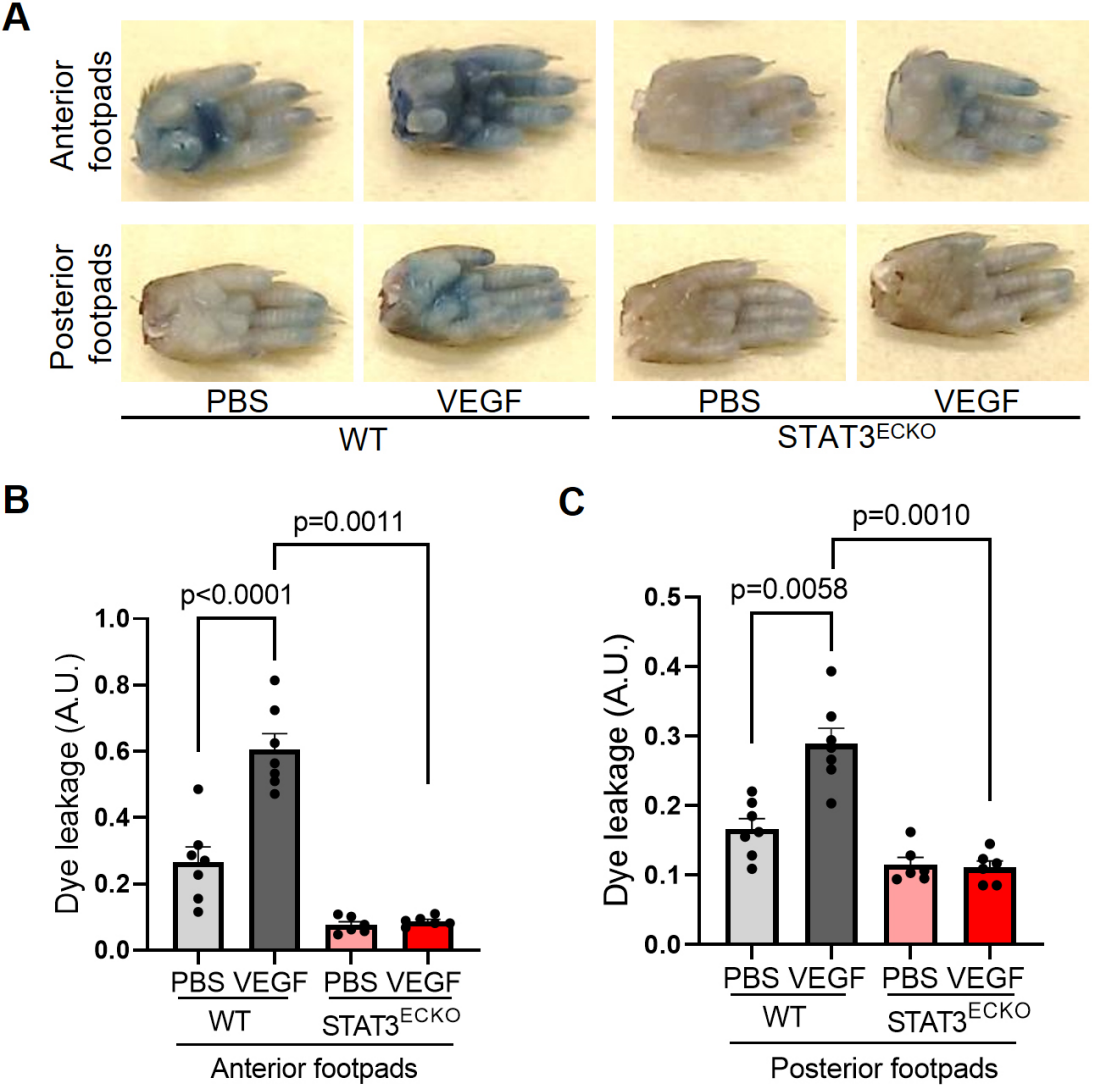
**Endothelial cell-specific STAT3 knockout mice exhibit decreased VEGF-induced permeability.** (A) Images of footpads from WT and endothelial cell-specific STAT3 knockout (STAT3^ECKO^) mice following tail vein injection with 1% Evans Blue dye and human recombinant VEGF-165 protein (2.5 µg/ml; left footpads) or PBS vehicle (right footpads) being injected into the root of the footpad. (B,C) Quantitation of Evans Blue leakage in Tie2-Cre negative; STAT3^flox/flox^ (WT) and Tie2-Cre positive; STAT3^flox/flox^ (STAT3^ECKO^) mice. *n*=7 mice in WT group and *n*=6 mice in STAT3^ECKO^ group. Each mouse was injected with PBS on the right anterior and posterior footpads and VEGF on the left anterior and posterior footpads. Multiple biological replicates were performed and depicted findings are representative. Mean±s.e.m., one-way ANOVA followed by Bonferroni test. A.U., arbitrary units.

To corroborate findings from the zebrafish vascular permeability model in a mammalian system, we standardized a mouse footpad permeability assay in endothelial cell-specific STAT3-deficient (STAT3^ECKO^) mice. Given that germline STAT3 deficiency leads to embryonic lethality in mice (Takeda et al., 1997), we generated STAT3^ECKO^ mice by crossing Tie2-Cre mice (Kisanuki et al., 2001) to STAT3 floxed mice (Moh et al., 2007) and confirmed STAT3 ablation in endothelium by observing a reduction in STAT3 via immunoblotting (Fig. S3). Prior reports have established that endothelium-specific STAT3 deficiency in mice does not impair vascular development or structure (Hoffmann et al., 2015; Kano et al., 2003; Miller et al., 2010; Wang et al., 2007a,b; Welte et al., 2003). To assess VEGF-induced vascular permeability, anesthetized STAT3^ECKO^ or corresponding control mice were injected intravenously with Evans Blue dye and subcutaneously injected with recombinant VEGF protein (2.5 µg/ml in PBS; left footpads) or vehicle (right footpads). After 30 min, footpads were excised from euthanized mice, and extravasated dye was extracted via formamide and measured by spectrometry to quantify VEGF-induced vascular permeability. We observed significantly decreased extravasation of Evans Blue dye in STAT3^ECKO^ mice relative to controls, suggesting that STAT3 is an important transducer of VEGF-induced vascular permeability (Fig. 2).

### Pharmacological inhibition of STAT3 stabilizes endothelial barrier integrity following VEGF stimulation

**Fig. 3.**
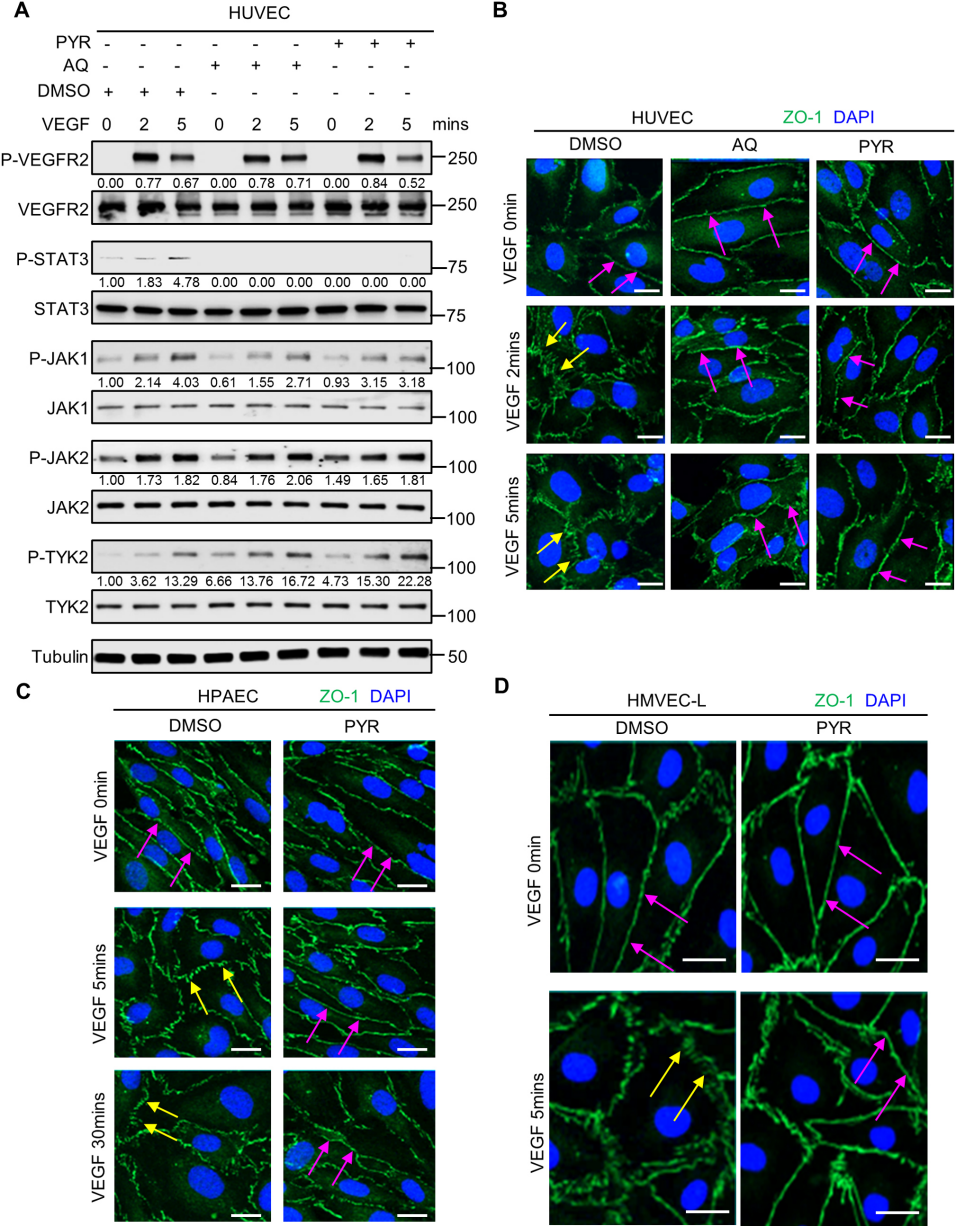
**Pharmacological inhibition of STAT3 stabilizes endothelial barrier integrity following VEGF stimulation in human endothelial cells.** (A) Serum-starved human umbilical vein endothelial cells (HUVECs) were pretreated with DMSO (vehicle control) for 1 h, 30 µM AQ for 4 h, or 10 µM PYR for 1 h prior to VEGF (25 ng/ml) stimulation for 0, 2 or 5 min. Lysates were immunoblotted. Densitometry was performed, and the values below the rows of bands represent the ratio of phosphorylated protein to respective total protein. (B) Human VEGF-165 recombinant protein (VEGF; 25 ng/ml) stimulation of HUVECs promotes ZO-1 (green) disorganization at endothelial cell junctions (yellow arrows; left column; DMSO vehicle control pretreatment for 1 h prior to VEGF stimulation). ZO-1 organization is maintained upon pretreatment with 30 μM AQ for 4 h (magenta arrows; middle column) or 10 μM PYR for 1 h (magenta arrows; right column) prior to VEGF stimulation. Nuclei were stained with DAPI (blue). (C) Serum-starved human pulmonary artery endothelial cells (HPAECs) were pretreated with 10 µM PYR for 1 h prior to VEGF (25 ng/ml) stimulation for 0, 5 or 30 min. VEGF stimulation promotes disorganization of ZO-1 (green) at endothelial cell junctions (yellow arrows). ZO-1 organization is maintained when HPAECs were pretreated with PYR (magenta arrows). Nuclei were stained with DAPI (blue). (D) VEGF (25 ng/ml) stimulation of human lung microvascular endothelial cells (HMVEC-Ls) promotes ZO-1 (green) disorganization at endothelial cell junctions (yellow arrows). ZO-1 organization is maintained upon pretreatment with 20 μM PYR for 6 h prior to VEGF stimulation (magenta arrows). Nuclei were stained with DAPI (blue). At least two biological replicates were performed for each experiment depicted in A-D. Scale bars: 20 µm.

Given that genetic ablation of STAT3 reduces vascular permeability in zebrafish and mice, we sought to test the effects of pharmacological STAT3 inhibition on endothelial barrier integrity. Pyrimethamine (PYR; Daraprim^®^) (Khan et al., 2018) and atovaquone (AQ; Mepron^®^) (Xiang et al., 2016) are US Food and Drug Administration (FDA)-approved antimicrobial agents that were recently discovered as new inhibitors of STAT3 activity. PYR was identified as a STAT3 inhibitor through a chemical-biology approach. AQ rapidly and specifically downregulates cell-surface expression of glycoprotein 130, which is required for STAT3 activation in multiple contexts. These compounds have been shown to be safe in humans because they inhibit STAT3 at concentrations routinely achieved in human plasma (Khan et al., 2018; Takakura et al., 2011; Xiang et al., 2016). To our knowledge, PYR and AQ have yet to be specifically assessed in endothelium, as prior studies have primarily evaluated their STAT3 inhibitory effects on tumor cells of epithelial origin (Cheng et al., 2020; Coates et al., 2020; Khan et al., 2018; Lin et al., 2018; Liu et al., 2019a,b; Wu et al., 2020; Xiang et al., 2016; Zhou et al., 2020). Therefore, we first treated HUVECs with various concentrations of PYR or AQ for different durations to determine the optimal conditions, while not exceeding the concentrations typically achieved in human plasma (Fig. S4). In serum-starved HUVECs treated with 10 µM PYR for 1 h or 30 µM AQ for 4 h followed by VEGF stimulation, we observed decreased phosphorylation of STAT3 at Y705 (Fig. 3A; Fig. S5). We confirmed activation of VEGF signaling by assessing the phosphorylation status and total protein levels of VEGFR-2 and JAK family members (Fig. 3A; Fig. S5). Given that VEGFR-2, JAK1, JAK2 and TYK2 are upstream of STAT3, PYR- and AQ-mediated STAT3 inhibition does not affect activation of these proteins. Taken together, these results suggest that PYR and AQ inhibit VEGF-induced STAT3 activation in human endothelial cells.

As vascular barrier stability is, in large part, regulated by intercellular junctions (Lampugnani, 2012), we examined whether STAT3 inhibition affects junctional organization in human endothelial cells. We first confirmed that PYR and AQ inhibit STAT3 activation, as evident by decreased phosphorylation of STAT3 at Y705 (Fig. 3A). As expected, the upstream activity of VEGFR-2 and JAK family members was unaffected by PYR and AQ (Fig. 3A). In HUVECs, human pulmonary artery endothelial cells (HPAECs) and human lung microvascular endothelial cells (HMVEC-Ls), we demonstrated, by immunofluorescence, that VEGF induces zonula occludens 1 (ZO-1; also known as TJP1) disorganization, indicative of tight junction instability (Fig. 3B-D). STAT3 pharmacological inhibition via PYR or AQ restores ZO-1 organization in HUVECs, suggesting that VEGF may mediate vascular permeability through STAT3-regulated control of ZO-1 (Fig. 3B). PYR pretreatment also prevented VEGF-induced ZO-1 disorganization in HPAECs and HMVEC-Ls cultured in standard conditions (Fig. 3C,D). Correspondingly, we demonstrate that C188-9, a high-affinity inhibitor of STAT3 that targets its phosphotyrosyl peptide binding site within the SRC homology 2 (SH2) domain (Bharadwaj et al., 2016), prevents VEGF-mediated ZO-1 disorganization in HUVECs and HPAECs (Fig. S6A,B). Similar to AQ and PYR, C188-9-mediated STAT3 inhibition does not affect activation of JAK2 (Fig. S6C). Testing multiple STAT3 inhibitors in three different types of human endothelial cells and consistently finding that STAT3 inhibition prevents VEGF-induced ZO-1 disorganization reinforces that STAT3 serves as a master regulator of vascular permeability in a variety of endothelial cell types.

**Fig. 4.**
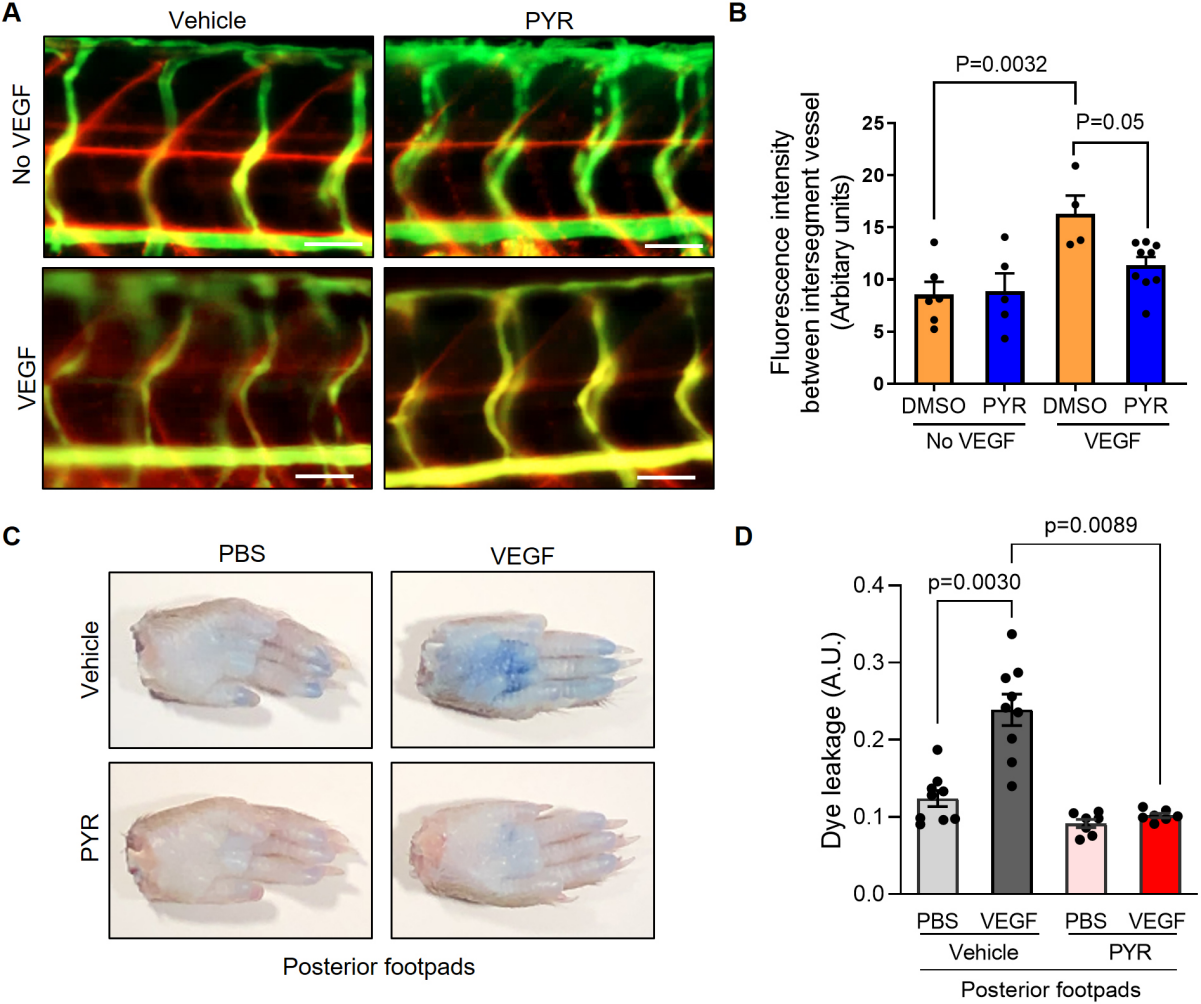
**Suppression of STAT3 activity by pyrimethamine (PYR) inhibits VEGF-induced vascular permeability in zebrafish and mice.** (A) Microangiography using 70 kDa Texas Red-dextran permeabilizing tracer (red) and 2000 kDa FITC-dextran intersegmental vessel marker (green) was performed on 3 dpf zebrafish without induced VEGF pretreated with DMSO (*n*=6) or 25 μM PYR (*n*=5) or 3 dpf zebrafish with induced VEGF pretreated with DMSO (*n*=4) or 25 μM PYR (*n*=9) for 3 days. Representative images shown were obtained using a Zeiss Apotome 2 microscope with a Fluar 5×/0.25 NA lens at RT. Scale bars: 50 μm. (B) The quantitative analysis of vascular permeability without VEGF stimulation or upon VEGF stimulation in zebrafish pretreated with DMSO or PYR. Mean±s.e.m., one-way ANOVA followed by Bonferroni test. (C) Representative images of footpads from mice treated with vehicle or PYR following tail vein injection with 1% Evans Blue and footpad injection of VEGF (2.5 μg/ml) or PBS vehicle. (D) Quantitation of Evans Blue dye leakage in C57BL/6 WT mice treated with vehicle or PYR. *n*=9 mice in the vehicle group and *n*=7 mice in the PYR group. Each mouse was injected with PBS in the right posterior footpad and VEGF in the left posterior footpad. Multiple biological replicates were performed and depicted findings are representative. Mean±s.e.m., one-way ANOVA followed by Bonferroni test.

Given that genetic ablation of STAT3 in zebrafish and mice reduces VEGF-mediated vascular permeability, we next sought to assess the effects of pharmacological STAT3 inhibition on endothelial barrier integrity *in vivo*. To that end, VEGF-inducible zebrafish embryos were exposed to 25 µM PYR via their water for 3 days, and vascular permeability was assessed using fluorescence microangiography. We observed decreased VEGF-induced vascular permeability in zebrafish treated with PYR relative to controls, suggesting that pharmacological inhibition of Stat3 reduces VEGF-induced vascular permeability in zebrafish (Fig. 4A,B; Fig. S7). To evaluate permeability upon STAT3 inhibition in mice, we treated wild-type C57BL/6 mice with 75 mg/kg PYR daily for 15 consecutive days, 100 mg/kg C188-9 daily for 7 consecutive days, or each corresponding vehicle control via intraperitoneal injection, followed by vascular permeability assessment, as described for similar experiments. We observed decreased VEGF-induced extravasation of dye in mice treated with STAT3 inhibitor relative to controls, suggesting that pharmacological inhibition of STAT3 reduces vascular permeability in mice (Fig. 4C,D; Fig. S6D).

### JAK2 activates STAT3 to promote VEGF/VEGFR-2-induced vascular permeability

**Fig. 5.**
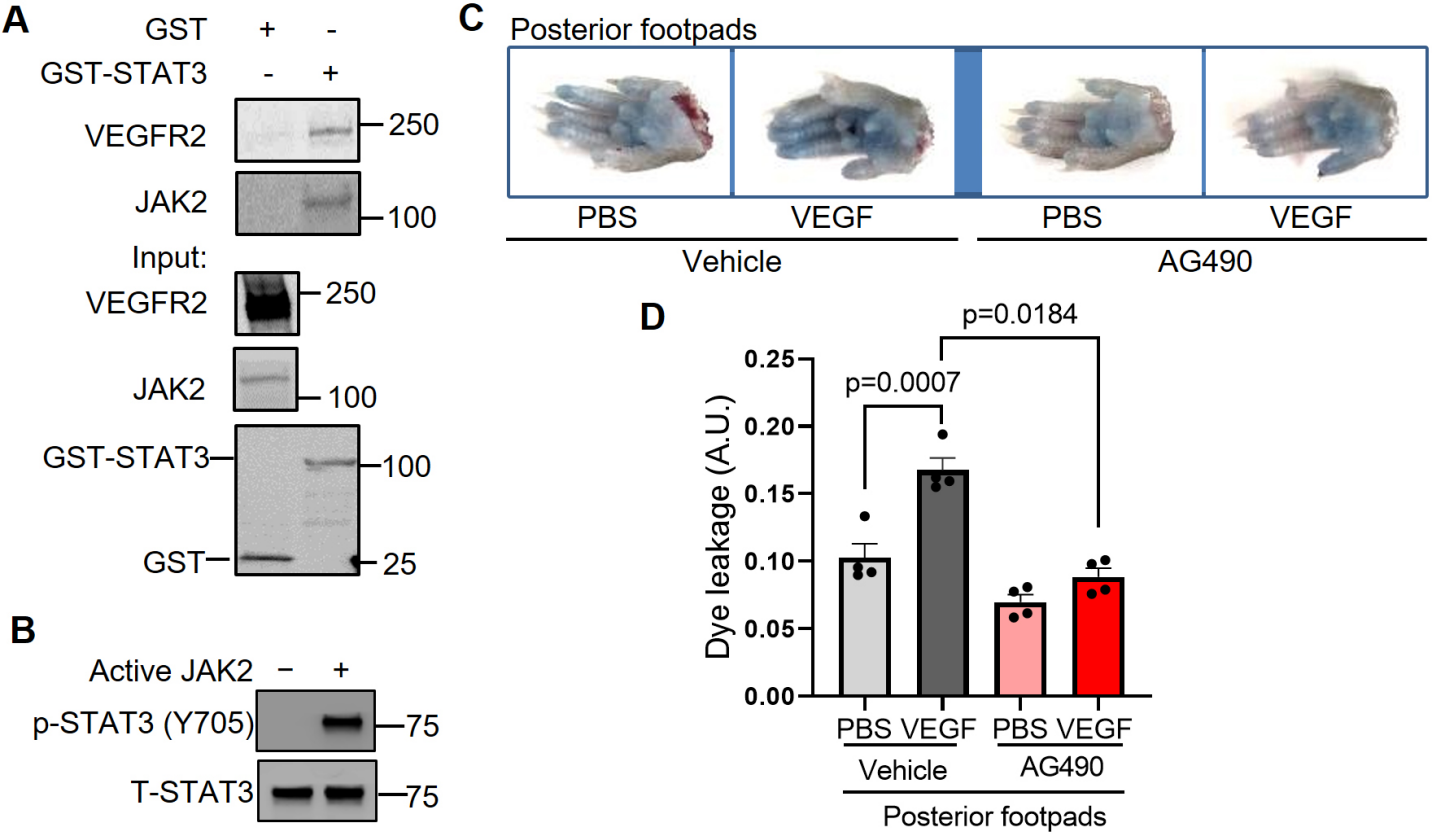
**JAK2 phosphorylates STAT3 to transduce VEGF/VEGFR-2 signaling and promote vascular permeability.** (A) To perform a STAT3 GST pull-down of VEGFR-2 and JAK2, lysates of HUVECs stimulated with serum for 30 min were used as prey. GST fusion protein STAT3 expressed in 293F cells was used as bait. GST alone served as a negative control. Binding experiments were analyzed by SDS-PAGE and visualized by immunoblotting. GST-STAT3 and GST were each detected using an anti-GST antibody. Three biological replicates were performed and depicted findings are representative. (B) JAK2 phosphorylates STAT3 *in vitro*. *In vitro* kinase assays were performed using purified human STAT3 protein and kinase active JAK2 protein. The results shown here are representative of two independent experiments. (C) Representative images of footpads from C57BL/6 WT mice treated with vehicle or JAK2 inhibitor AG490. Following tail vein injection with 1% Evans Blue dye, human VEGF-165 protein (2.5 μg/ml) or PBS vehicle was injected into the root of the footpad. After 30 min, the mice were euthanized and the footpads were excised. (D) Quantitation of Evans Blue dye leakage in C57BL/6 mice treated with vehicle or AG490. *n*=4 mice per group. Each mouse was injected with PBS in the right posterior footpad and VEGF in the left posterior footpad. Two biological replicates were performed and depicted findings are representative. Mean±s.e.m., one-way ANOVA followed by Bonferroni test.

Phosphorylation of STAT3 at Y705 causes its dimerization, nuclear translocation and DNA binding to control transcription of genes, including regulators of vascular permeability. The kinase(s) that phosphorylates STAT3 Y705 in endothelial cells has not been well identified. VEGF signals through VEGFR-2, which possesses intrinsic kinase activity through which it may activate STAT3 directly or through another kinase, such as JAK1, JAK2, JAK3, TYK2 or SRC (Bartoli et al., 2003; Chen et al., 2008; Yahata et al., 2003; Zhao et al., 2015). Here, our findings suggest that JAK2 phosphorylates STAT3 in HUVECs and that JAK2 is required for VEGF/VEGFR-2-mediated vascular permeability *in vivo*. Specifically, we first tested whether JAK2 physically interacts with STAT3. Indeed, in glutathione S-transferase (GST) pull-down assays, purified GST-STAT3 fusion protein associates with each of VEGFR-2 and JAK2 present in lysates derived from stimulated HUVECs (Fig. 5A). To determine whether JAK2 directly phosphorylates STAT3, we performed an *in vitro* kinase assay using kinase active JAK2 protein with STAT3 protein we purified from Sf9 insect cells and found that JAK2 phosphorylates STAT3 at Y705 (Fig. 5B). To test the role of JAK2 in the regulation of vascular barrier integrity, we administered a JAK2 inhibitor, AG490, to C57BL/6 mice daily for 7 consecutive days and assessed VEGF-induced vascular permeability of intravenously injected Evans Blue dye. Pharmacological inhibition of JAK2 substantially reduced VEGF-mediated vascular permeability in mice (Fig. 5C,D), suggesting that vascular permeability induced by VEGF/VEGFR-2 may require JAK2. Given that AG490 can also inhibit EGF and JAK3, additional studies are necessary to rule out their potential involvement in signaling cascades that lead to STAT3 phosphorylation. Likewise, it is also possible that JAK2-mediated, STAT3-independent mechanisms contribute to VEGF-induced vascular leakage. However, our collective findings suggest that JAK2 activates STAT3 via Y705 phosphorylation to promote VEGF/VEGFR-2-induced vascular permeability.

### STAT3 transcriptionally activates ICAM-1, a cell adhesion molecule that promotes vascular permeability

**Fig. 6.**
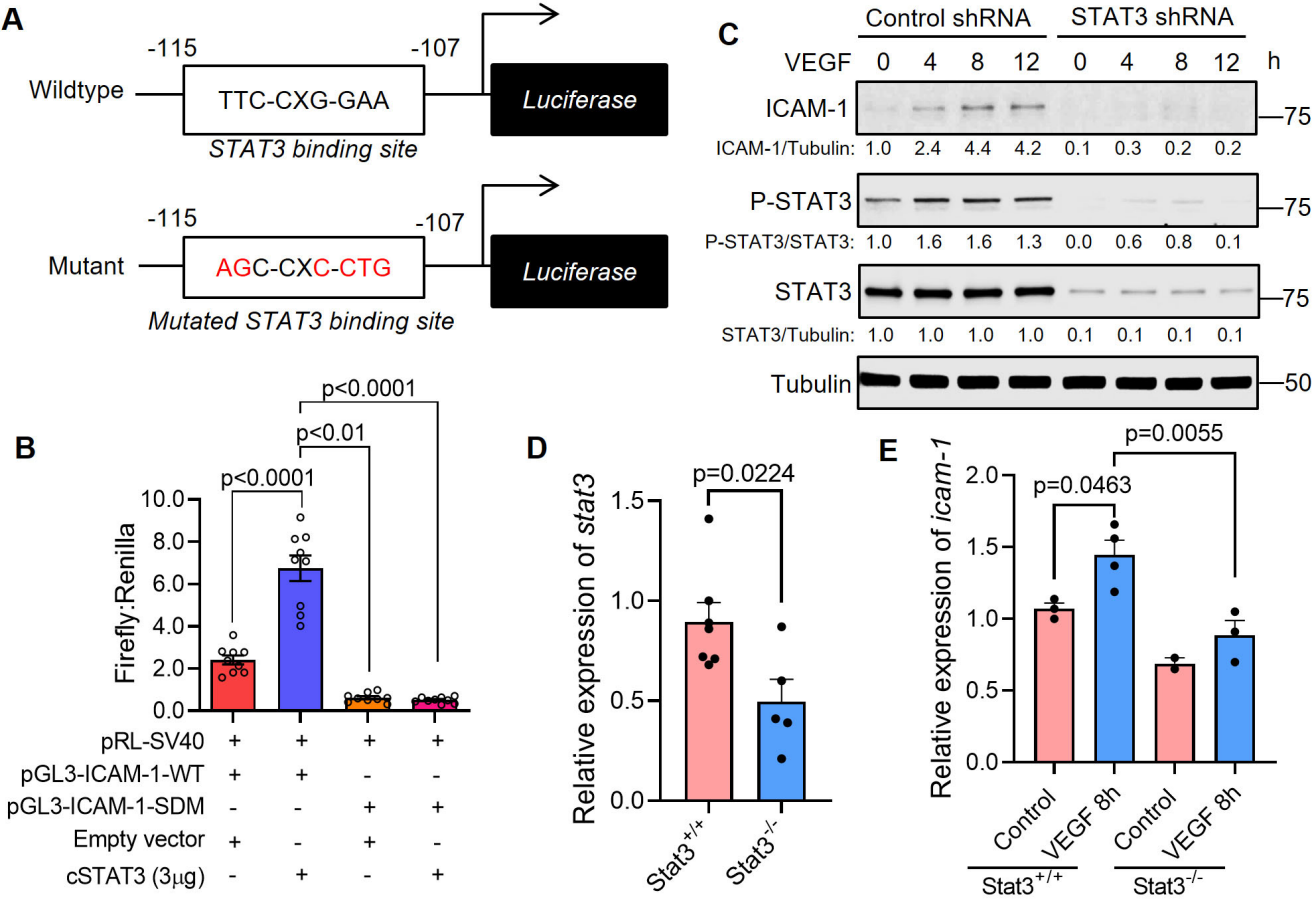
**STAT3 transcriptionally activates ICAM-1, a cell adhesion molecule that promotes vascular permeability.** (A) Top: the pGL3-ICAM1-WT plasmid containing the human ICAM-1 promoter with a STAT3 binding site located at −115 to −107 bp. Bottom: the pGL3-ICAM1-SDM plasmid with a site-directed mutation (SDM) in the STAT3 binding site as indicated. (B) Dual luciferase assays were performed in HUVECs that were transfected with pGL3-ICAM1-WT or pGL3-ICAM1-SDM and empty vector or constitutively active STAT3. Firefly and Renilla luminescence was measured and plotted as a ratio. Mean±s.e.m., one-way ANOVA followed by Bonferroni test. *n*=9 technical replicates. Depicted findings are representative of three independent experiments. (C) HUVECs that had been stably transduced with lentivirus encoding STAT3-specific shRNA or control shRNA were stimulated with human VEGF-165 protein (25 ng/ml) and the lysates were immunoblotted for ICAM1, p-STAT3 (Y705) and total STAT3. Depicted data are representative of three biological replicates. (D) RNA was harvested from VEGF; Stat3^+/+^ or VEGF; Stat3^−/−^ 3 dpf embryos for quantitative PCR. *stat3* transcripts are reduced in VEGF; Stat3^−/−^ (*n*=5) compared to VEGF; Stat3^+/+^ zebrafish (*n*=7). Mean±s.e.m., unpaired, two-tailed Student's *t*-test. (E) The expression of *icam-1* was assessed by real-time quantitative PCR using RNA derived from each zebrafish embryo in the absence of VEGF induction (Stat3^+/+^, *n*=3; Stat3^−/−^, *n*=2) or 8 h following VEGF induction (Stat3^+/+^, *n*=4; Stat3^−/−^, *n*=3) in the heat-inducible VEGF; Stat3 mutant zebrafish. Mean±s.e.m., one-way ANOVA followed by Bonferroni test.

We next sought to investigate the molecular mechanisms through which STAT3 transcriptionally regulates VEGF-induced vascular permeability. To that end, we identified a STAT3 binding site within the promoter region of intercellular adhesion molecule 1 (ICAM-1) (Fig. 6A). ICAM-1 is a cell surface glycoprotein that has been linked to regulation of VEGF-induced vascular permeability (Miyamoto et al., 2000) and known to be activated by cytokines, including IFNγ, IL-6 and TNFα (also known as TNF) (Han et al., 2016; Park et al., 2013). Thus, we hypothesized that one way STAT3 regulates VEGF-induced vascular permeability is through transcriptional control of ICAM-1. Using luciferase-based reporter assays, we demonstrate increased ICAM-1 promoter activity upon co-transfection with constitutively active *STAT3* cDNA plasmid (Fig. 6B). Mutation of the STAT3 binding site within the ICAM-1 promoter region prevents activation of the ICAM-1 promoter by constitutively active STAT3 (Fig. 6B). ICAM-1 protein is upregulated following VEGF-mediated activation of STAT3 in human endothelial cells, and VEGF-mediated induction of ICAM-1 protein can be ablated by stable silencing of STAT3 via lentiviral transduction of STAT3-specific shRNA (Fig. 6C; Fig. S8). Correspondingly, in Stat3^+/+^ zebrafish, the expression of *icam-1* (also known as *sc:d0202*) increased after VEGF induction, whereas this VEGF-dependent *icam-1* upregulation was not observed in Stat3^−/−^ zebrafish (Fig. 6D,E). Taken together, our findings suggest that VEGF-induced STAT3 transcriptionally regulates ICAM-1 in human endothelium cells and *in vivo*.

## DISCUSSION

Despite the prominent role of VEGF-induced vascular permeability in pathogenesis, a greater collective molecular understanding of its regulatory mechanisms is necessary to therapeutically improve vascular barrier integrity to prevent subsequent edema and tissue damage in vascular diseases like myocardial infarction, ischemic stroke and acute lung injury. These research efforts have been hindered by limitations and inconsistencies in currently accessible models. Endothelial cell culture models often fail to uniformly replicate *in vivo* models of hyperpermeability (Curry, 1992; Kevil et al., 1998). Molecular regulation of vascular barrier integrity can vary greatly based on various pathophysiological contexts and differences in cell types, tissues, anatomical locations and species (Bates, 2010). To overcome these obstacles, we took advantage of a transgenic VEGF-inducible zebrafish model that is amenable to genetic manipulation and reproducible live imaging of vascular permeability in optically clear zebrafish embryos using fluorescently labeled tracers (Hoeppner et al., 2012, 2015). Additionally, the widespread recent emergence of CRISPR/Cas9 genome-editing techniques has enhanced the study of genetic regulators of endothelial barrier function in genetically engineered zebrafish mutants.

In response to a variety of cytokines, growth factors and hormones, STAT proteins are activated via phosphorylation, dimerize, translocate to the nucleus, and bind to specific target gene promoters to regulate cellular processes, such as proliferation, differentiation, migration and survival (Rawlings et al., 2004). Reports have demonstrated that VEGF rapidly induces STAT3 tyrosine phosphorylation and nuclear translocation in microvascular endothelial cells (Bartoli et al., 2003; Chen et al., 2008; Huang et al., 2016; Yahata et al., 2003). A positive-feedback loop exists, as VEGF-induced STAT3 has been shown to be a direct transcriptional activator of the VEGF promoter (Niu et al., 2002; Wei et al., 2003). Functionally, phosphorylation of STAT3 by VEGF/VEGFR-2 signaling is required for endothelial cell migration (Yahata et al., 2003). Correspondingly, it was recently reported that VEGFR-2 phosphorylation is necessary for vascular permeability in mouse models of retinopathy (Smith et al., 2020). Here, we show the integral role of STAT3 in the molecular regulation of vascular permeability using VEGF-inducible zebrafish crossed to CRISPR/Cas9-generated Stat3 mutants. Genomic ablation of Stat3 substantially reduces VEGF-mediated extravasation of fluorescent dextran from intersegmental vessels within the trunk region of larval zebrafish. Correspondingly, genetic knockout of STAT3 in the endothelium of mice increases vascular barrier integrity. Pharmacological inhibition of STAT3 using multiple inhibitors reduces VEGF-mediated vascular permeability in wild-type mice and prevents tight junction disorganization typically caused by VEGF stimulation of cultured human endothelial cells. VEGF/VEGFR-2 signaling results in JAK2-mediated activation of STAT3, which enables STAT3 to translocate to the nucleus and transcriptionally regulate genes involved in vascular barrier integrity, including *ICAM-1*. Here, we demonstrate that *ICAM-1* is a target of STAT3 transcriptional regulation. ICAM-1, a cell surface glycoprotein, has been shown to mediate VEGF-induced vascular permeability and leukostatsis (Miyamoto et al., 2000). IFNγ stimulation induces expression of both membrane-associated and soluble forms of ICAM-1, the latter of which binds to lymphocyte function associated antigen-1 (LFA-1; also known as ITGAL) to regulate immunomodulation (Budnik et al., 1996). Mechanistically, IFNγ and other pro-inflammatory cytokines induce expression of CCL15, which stimulates expression of ICAM-1 via STAT3-mediated transcriptional activation of the ICAM-1 promoter (Park et al., 2013; Yuan et al., 1994). ICAM1 is upregulated during inflammation stimulated by NF-κB or TNFα. In a rat lung injury model, ICAM-1 was suppressed via inhibition of TNFα- and IL-6-induced JAK2/STAT3 activation through dexamethasone treatment (Han et al., 2016). Taken together with previous reports, our data suggest that VEGF-induced STAT3 transcriptionally regulates ICAM-1 to control vascular permeability.

Although genomic STAT3 deficiency in mice results in embryonic lethality, the endothelium tissue-specific STAT3 knockout mice we report here are healthy and fertile with no overt defects in vascular development or structure, which coincides with previous findings (Hoffmann et al., 2015; Kano et al., 2003; Miller et al., 2010; Wang et al., 2007a,b; Welte et al., 2003). Correspondingly, we observe normal vascular development in CRISPR/Cas9-generated zebrafish with homozygous genomic Stat3 deficiency, visualized by fluorescence microangiography (Fig. 1B). *In vitro* studies suggest that a dominant-negative form of STAT3 suppresses human dermal microvascular endothelial cell tube formation on Matrigel and collagen (Yahata et al., 2003). However, endothelial cells isolated from endothelium-specific STAT3 knockout mice and cultured *ex vivo* initiate normal tube formation (Kano et al., 2003). Although endothelial cell-specific STAT3 knockout mice undergo physiologically normal developmental angiogenesis, these mice exhibit defects in tissue repair and decreased recovery from vascular injuries, including myocardial infarction, cerebral ischemia and ischemia-reperfusion injuries (Hoffmann et al., 2015; Wang et al., 2007a,b). These observations highlight the need to understand the temporal dynamics through which STAT3 regulates pathological vascular permeability and edema as well as restorative angiogenesis to repair damaged tissue. In regards to these temporal dynamics, we observe STAT3-mediated changes in VEGF-induced vascular permeability within the range of 5-30 min in zebrafish, mice and cultured endothelial cells, suggesting that STAT3 may have direct effects on permeability. We also show that STAT3 transcriptionally regulates target genes involved in vascular permeability, such as ICAM-1, over the course of hours. However, additional studies beyond the scope of this report are necessary to more precisely define how STAT3 directly and indirectly (i.e. transcriptional regulation) controls VEGF-induced vascular permeability.

The functional role of STAT3 in VEGF-induced permeability has not been directly investigated. Prior studies suggest that other permeability inducers – such as IL-6, IgG, IgE, histamine, lipopolysaccharides and eotaxin – mediate vascular permeability through STAT3 signaling (Alsaffar et al., 2018; Hox et al., 2016; Jamaluddin et al., 2009; Lee et al., 2012; Tang et al., 2011; Wei et al., 2013; Yeh et al., 2006; Yun et al., 2017). For example, an *in vitro* study using human endothelial cells recently demonstrated that IL-6 promotes sustained loss of endothelial barrier function via STAT3 signaling (Alsaffar et al., 2018), and IL-6-induced STAT3 activation has been shown to induce vascular permeability in ovarian endothelial cells (Wei et al., 2013). Furthermore, IL-6-induced retinal endothelial permeability was found to be dependent upon STAT3 activation in mouse retina (Yun et al., 2017). Dominant-negative STAT3 has been shown to reduce IL-6-induced vascular permeability associated with malignant pleural effusion in lung adenocarcinoma (Yeh et al., 2006) and decrease IgG-mediated vascular permeability during acute lung injury (Tang et al., 2011). Patients and mice harboring STAT3 mutations, which cause autosomal dominant hyper-IgE syndrome (AD-HIES), have been shown to be partially protected from anaphylaxis. HUVECs derived from patients with AD-HIES or treated with a STAT3 inhibitor exhibit decreased histamine- and IgE-mediated leakage (Hox et al., 2016). Inhibition of STAT3 phosphorylation decreases lipopolysaccharide-induced myocardial vascular permeability in a murine model (Lee et al., 2012). Eotaxin stimulates STAT3 phosphorylation and disrupts human endothelial barrier integrity (Jamaluddin et al., 2009). Taken together with the important role of STAT3 in modulating VEGF-mediated vascular permeability in the vertebrate models presented here, studies collectively suggest that STAT3 is a central regulator of vascular barrier function.

Considerable effort has been devoted to the development of STAT3 inhibitors as therapeutic agents. In addition to the important role of STAT3 in the pathogenesis of vascular disease, ischemia and other tissue injuries, STAT3 is aberrantly overexpressed in many human tumor types and correlates with poor cancer prognoses. STAT3 peptide inhibitors designed to target the p-Y-peptide binding pocket within the SH2 domain stemmed from elucidating the STAT3β homodimer structure have been developed (Ren et al., 2003; Shao et al., 2006, 2004; Turkson et al., 2001). However, the clinical applicability of these STAT3 peptide inhibitors has been hindered by their lack of stability and inability to cross membranes. Non-peptidic small molecule inhibitors of STAT3 exhibited promising *in vivo* activity in preclinical studies, but most of these STAT3 inhibitors failed to progress to clinical trials because they required medium-to-high micromolar concentrations to achieve sufficient activity and necessitated additional optimization in order to be systemically administered to human subjects (Bhasin et al., 2008). Although developing and translating STAT3 inhibitors to the clinic has proven difficult because STAT3 is a transcription factor without intrinsic enzymatic activity (Wong et al., 2017), several compounds that inhibit either the function or expression of STAT3 are currently in clinical trials (Johnson et al., 2018), including a decoy oligonucleotide that competitively inhibits STAT3 interactions with its target gene promoter elements (Sen et al., 2012) and an antisense oligonucleotide inhibitor of STAT3 expression, AZD9150 (Reilley et al., 2018).

PYR is a clinically available, FDA-approved agent that directly inhibits STAT3-dependent transcription. This antimicrobial drug was discovered as a new STAT3 inhibitor based on its ability to oppose the gene expression signature of STAT3 (Khan et al., 2018; Takakura et al., 2011). PYR inhibits STAT3 phosphorylation and transcriptional activity at micromolar concentrations known to be routinely achieved in humans without toxicity (Khan et al., 2018). Our study results indicate that PYR suppresses STAT3 activation in endothelial cells, as we observed decreased phosphorylation of STAT3 Y705. PYR treatment prevents VEGF-induced ZO-1 disorganization, which suggests that STAT3 inhibition improves tight junction instability in endothelium. We demonstrate that PYR administered to zebrafish and mice substantially reduces VEGF-induced vascular permeability. AQ, another FDA-approved antimicrobial agent with a strong human safety profile, has been shown to rapidly and specifically downregulate cell-surface expression of glycoprotein 130, which is required for STAT3 activation in multiple contexts (Xiang et al., 2016). Like PYR, we show that AQ stabilizes endothelial tight junctions in the presence of VEGF stimulation. We also show that the STAT3 peptide inhibitor, C188-9, reduces VEGF-induced vascular permeability in mouse models and cultured human endothelial cells. Our collective studies using three different STAT3 inhibitors suggest that suppression of STAT3 activity protects the endothelial barrier from VEGF-mediated vascular permeability. However, it is important to consider that STAT3 may also regulate changes in genes that control vascular permeability in a VEGF-independent manner. In support of this notion, endothelial cells were treated with PYR for 4 h or AQ for 1 h before VEGF stimulation, such that VEGF-independent gene expression changes occurring during STAT3 inhibitor treatment may contribute to the reduced vascular permeability observed as decreased endothelial cell junction disorganization (Fig. 3). That said, we also observed similar reductions in endothelial cell junction disruption in endothelial cells treated with C188-9 for only 5 min prior to VEGF treatment, suggesting that VEGF-dependent phosphorylation of STAT3 contributes to vascular permeability (Fig. S6). Our work underscores the importance of STAT3 as a central mediator of vascular permeability and emphasizes the need for additional research to precisely dissect VEGF-dependent and -independent effects of STAT3 on vascular permeability. Future studies testing compounds that inhibit STAT3 activity, particularly PYR and AQ, given their clinical accessibility, in models of human diseases involving pathological vascular permeability are warranted.

## MATERIALS AND METHODS

### Cell culture

HUVECs, HPAECs and HMVEC-Ls were cultured in plates that had been pretreated for 30 min with collagen I (354231, Corning). Human endothelial cells were certified prior to purchase from Lonza, used exclusively at low passages and authenticated by morphological inspection. HUVECs were maintained in EBM Endothelial Cell Growth Basal Medium (CC-3121, Lonza) supplemented with EGM Endothelial Cell Growth Medium SingleQuots (CC-4143, Lonza). HPAECs were cultured in EBM-2 Basal Medium (CC-3156, Lonza) supplemented with EGM SingleQuots (CC-4176, Lonza). HMVEC-Ls were cultured in EBM-2 Basal Medium (CC-3156, Lonza) supplemented with EGM SingleQuots (CC-4147, Lonza). For *in vitro* studies, HUVECs, HPAECs or HMVEC-Ls grown in culture to ∼80% confluence were serum starved for 16 h and subsequently stimulated with human recombinant VEGF-165 protein (25 ng/ml; R&D Systems; MNPHARM) for indicated durations. When applicable, cells were treated with inhibitors or control vehicle for various indicated periods of time following serum starvation and preceding VEGF-165 protein stimulation.

### Antibodies

Immunoblotting was performed using antibodies purchased from Cell Signaling Technology (CST) to detect ZO-1 (13663; 1:1000), phosphorylated VEGFR-2 (Tyr1175, 2478; 1:1000), phosphorylated STAT3 (Tyr705, 9145; 1:500), phosphorylated JAK2 (Tyr 1007/1008, 3771; 1:500), phosphorylated JAK1 (Tyr 1034/1035, 3331S; 1:500), phosphorylated TYK2 (Tyr1054/1055, 9321S; 1:500), VEGFR-2 (2479; 1:1000), STAT3 (12640; 1:1000), JAK2 (3230; 1:500), JAK1 (3332; 1:500) and TYK2 (14193; 1:500). Additional antibodies were obtained from Santa Cruz Biotechnology to detect phosphorylated STAT3 (Tyr705, sc-8059; 1:500), ICAM-1 (sc-18853; 1:500) and Tubulin (sc-5286; 1:500). The monoclonal antibody for detecting GST (MA4-004; 1:1000) was purchased from Thermo Fisher Scientific. The monoclonal antibody against phospho-Stat3 (Tyr708, D128-3; 1:1000) in zebrafish was obtained from MBL International Corporation. The polyclonal antibody against Cofilin (95057-142; 1:1000) was purchased from VWR. Horseradish peroxidase-conjugated anti-rabbit (7074; 1:5000) and anti-mouse (7076; 1:5000) secondary antibodies (1 μg/μl) were purchased from Cell Signaling Technology.

Immunofluorescence was performed using antibodies against ZO-1 (13663, CST; 1:200) or STAT3 (12640, CST; dilution 1:100). The cells were then washed with PBS and incubated with CF™ 488A goat anti-rabbit IgG secondary antibody (SAB4600389, Sigma-Aldrich; 1:1000) or Alexa Fluor 568 goat anti-rabbit IgG secondary antibody (A11036, Invitrogen; 1:1000).

FITC-conjugated rat anti-mouse CD31 antibody (553372, BD Biosciences; 1:1000) was used for sorting mouse primary endothelial cells based on green fluorescence.

### Immunoblotting

Human endothelial cells or 3 dpf zebrafish embryos with the yolk sac removed were lysed in RIPA buffer (Millipore) containing protease inhibitors (Roche) and phosphatase inhibitors (Sigma-Aldrich). Proteins were separated via 4-20% gradient SDS-PAGE (Bio-Rad), transferred to membranes, blocked with 5% bovine serum albumin (Sigma-Aldrich), and incubated with primary and secondary antibodies as described previously (Alam et al., 2018). Antibody-reactive protein bands were detected by enzyme-linked chemiluminescence (Thermo Fisher Scientific) using an ImageQuant^TM^ LAS 4000 instrument (GE Healthcare) and quantified using ImageJ software (Version 1.8.0_112; https://imagej.nih.gov/ij) to obtain densitometry values.

### Immunoprecipitation

HUVECs were lysed in RIPA buffer (Millipore) supplemented with protease inhibitor cocktail (Roche) and phosphatase inhibitor cocktail set V (Sigma-Aldrich). After quantifying protein using the Quick Start^TM^ Bradford protein assay (Bio-Rad), 500 µg protein lysate was loaded to the supplied spin column and immunoprecipitation was achieved following the manufacturer's protocol (Catch and Release Immunoprecipitation Kit, 17-500, Millipore).

### Immunofluorescence

HUVECs, HPAECs or HMVEC-Ls were seeded at 4×10^4^ cells per well onto EMD Millipore Millicell EZ Slides (PEZGS0416, Sigma Millipore), grown in complete medium for 48 h and subsequently serum starved for 16 h. After receiving inhibitors and/or human recombinant VEGF-165 stimulation, cells were fixed in 4% paraformaldehyde (Boston Bioproducts), permeabilized in cold methanol (Thermo Fisher Scientific), and immunofluorescence was performed. Nuclei were stained using 4′, 6-diamidino-2-phenylindole (DAPI; 8961S, Cell Signaling Technology). All images were captured on a Zeiss Apotome 2 microscope using a 20×/0.8 NA Plan Apochromat objective magnifying 2× to 4× and processed using ImageJ software.

### Vascular permeability assay in mice

Mice with endothelium-specific knockout of STAT3 were created by breeding transgenic mice STAT3^flox/flox^ (*Stat3*^tm1Xyfu^/J: The Jackson Laboratory) with Tg(Tek-cre)1Ywa/J mice (The Jackson Laboratory). C57BL/6 wild-type mice (027, Charles River Laboratories) were purchased and bred. Eight- to 10-week-old pathogen-free male and female mice housed in a temperature-controlled room with alternating 12-h light/dark cycles and fed a standard diet were used for experiments. To assess vascular permeability, Evans Blue dye (100 µl; 1% in PBS; VWR) was intravenously injected into the lateral tail vein of mice. After 15 min, mice were anesthetized with one dose of ketamine (90-120 mg/kg)/xylazine (5-10 mg/kg) via intraperitoneal injection. Depth of anesthesia was monitored by toe pinch, tail pinch and visual observation of respiration rate. Human recombinant VEGF-165 protein (2.5 µg/ml in PBS; MNPHARM; 20 µl total volume; left footpads) and PBS vehicle control (20 µl; right footpads) were each injected into one anterior and one posterior footpad. After 30 min, mice were euthanized using asphyxiation by CO_2_ inhalation to effect with a flow rate displacing less than 30% of the chamber volume per minute in accordance with Institutional Animal Care and Use Committee (IACUC) euthanasia guidelines and consistent with recommendations of the Panel of Euthanasia of the American Veterinary Medical Association. Following euthanasia, footpads were excised. Dye was extracted by incubation in formamide at 63°C overnight and quantified by spectroscopic detection at 620 nm using a Synergy Neo2 instrument (BioTek). The care and use of mice as well as all mouse experiments complied with all relevant institutional and national animal welfare laws, guidelines and policies, in accordance with protocols approved by the University of Minnesota Institutional Animal Care and Use Committee (IACUC).

### Assessment of vascular permeability in VEGF-inducible, Stat3-deficient zebrafish

Zebrafish were maintained in 28.5°C aquatic system water. The care and use of zebrafish as well as all zebrafish experiments complied with all relevant institutional and national animal welfare laws, guidelines and policies, in accordance with protocols approved by the University of Minnesota IACUC. Heat-shock-inducible VEGF transgenic zebrafish (i.e. *mn32Tg*; https://zfin.org/action/feature/view/ZDB-ALT-140213-2) were previously generated by co-injecting 2 nl pkTol2-h70-mC-hVEGF-gcG plasmid (12.5 ng/µl) and transposase mRNA (12.5 ng/µl) into one-cell-stage WT zebrafish embryos. Potential founders were selected by expression of enhanced green fluorescent protein (EGFP) in their eyes and raised to adulthood to produce F1 embryos for identification of true transgenic zebrafish (Hoeppner et al., 2012). Stat3-deficient zebrafish were previously generated by co-injecting 150 pg Cas9 mRNA and 50 pg guide RNA targeting 5′-GGTGCTGCTTGATGCGCCGC-3′ into NHGRI-1 zebrafish embryos. Mutation was detected by fluorescent-based PCR fragment analysis. Five nucleotides in exon 3 of the *stat3* gene, GGCGC, were deleted, resulting in a premature stop leading to a truncated protein (Pei et al., 2018). Transgenic VEGF-inducible zebrafish (Hoeppner et al., 2012) were outcrossed to zebrafish heterozygous for Stat3 deficiency (Stat3^+/−^) generated by CRISPR/Cas9 (Pei et al., 2018). Subsequently, VEGF-inducible; Stat3^+/−^ zebrafish were incrossed, and the one-cell-stage embryos were microinjected with 1.5 nl Cre mRNA (12.5 ng/µl) to excise the floxed mCherry cassette such that the *VEGF* transgene could be heat induced. Zebrafish expressing the VEGF-inducible transgene were identified by the presence of EGFP in their eyes using fluorescent imaging. At 2 dpf, 37°C heat shock of EGFP^+^-eyed zebrafish carrying the *VEGF* transgene was performed to confirm *VEGF* transgene activity via the absence of mCherry fluorescence. At 3 dpf, zebrafish were anesthetized and fluorescent microangiography was performed. A microneedle was inserted through the pericardium directly into the ventricle, and a mixture of 2000 kDa FITC-dextran and 70 kDa Texas Red-dextran (2 mg/ml in embryo water; Life Technologies) was injected. Immediately prior to imaging, 37°C heat shock induction of the *VEGF* transgene was performed for 10 min. Real-time imaging using SCORE methodology (Petzold et al., 2010) was performed using a Zeiss Apotome 2 microscope.

### Quantitation of zebrafish vascular permeability

ImageJ software was used to quantitate the extent of Texas Red-dextran in the extravascular space. Mean gray value was measured after converting the image type to 8-bit gray, setting scale to pixels, inverting the image and identifying four areas of extravascular space in the middle of the zebrafish trunk region using drawing/selection tools to avoid intersegmental vessels evident by green fluorescent signal originating from FITC-dextran. The background gray value minus the average gray value of the four regions was used for statistical analysis.

### Genotyping and validation of knockout efficiency

For mice, genomic DNA was extracted from a 2 mm tail biopsy excised from a 3-week-old mouse by adding 100 µl 50 mM NaOH and heating the sample at 95°C for 30 min, before cooling it down at 4°C for 30 min and neutralizing the solution by adding 10 µl 1 M Tris-HCl, pH 7.4. Primer pairs for genotyping the *Stat3*^tm1Xyfu^/J mice are forward primer 5′-TTGACCTGTGCTCCTACAAAAA-3′ and reverse primer 5′-CCCTAGATTAGGCCAGCACA-3′. The genotyping primer pairs for Tg(Tek-cre)1Ywa/J mice are forward primer 5′-CGCATAACCAGTGAAACAGCATTGC-3′ and reverse primer 5′-CCCTGTGCTCAGACAGAAATGAGA-3′.

To test the knockout efficiency of STAT3, mouse primary cells were harvested and pooled from the lung tissues of four wild-type (Tie2 Cre-, STAT3^flox/flox^) mice or four STAT3^ECKO^ (Tie2 Cre^+^, STAT3^flox/flox^) mice and incubated in FITC-conjugated rat anti-mouse CD31 antibody solution followed by sorting based on green fluorescence. Collected primary endothelial cells were lysed in RIPA buffer supplemented with proteinase inhibitor for subsequent immunoblotting using antibodies against STAT3 and Tubulin.

For zebrafish, each individual zebrafish was euthanized, and the tail was cut with a sterile scalpel distal to the intestine under a stereomicroscope (Dupret et al., 2018). Genomic DNA was extracted using standard techniques by adding 10 µl 50 mM NaOH to the individual zebrafish or the tail biopsy and heating the sample at 95°C for 30 min, before cooling it down at 4°C for 30 min and neutralizing the solution by adding 1 µl 1 M Tris-HCl, pH 7.4. For subsequent *stat3* genotyping, we performed PCR using the primers 5′-GGTCTTCCACAACCTGCTG-3′ and 5′-TAGACGCTGCTCTTCCCAC-3′ and then purified PCR products with a DNA Clean & Concentrator-5 kit (D4003, Zymo/Genesee Scientific) before performing Sanger sequencing via Genewiz.

To verify that Stat3 is ablated in Stat3^−/−^ fish, the anterior part of the zebrafish was homogenized with a pestle in 1× Laemmli buffer supplemented with protease inhibitor (Roche) followed by centrifugation at 20,800 ***g*** for 10 min at 4°C. The protein recovered from the supernatant was used for immunoblotting using antibodies against Stat3 and Tubulin.

### Real-time quantitative PCR

The anterior part of the zebrafish embryo was transferred to a 1.5 ml Eppendorf tube and 200 μl Trizol was added. Total RNA was extracted by mashing the sample with a pestle, rinsing the pestle with 100 μl Trizol, adding 60 μl chloroform and collecting the supernatant after centrifugation at 800 ***g*** for 15 min at 20°C followed by purifying RNA according to the RNA cleanup protocol provided in the RNeasy Mini Kit (Qiagen).

Real-time quantitative PCR was performed on a QuantStudio 12K Flex Real-Time PCR system (Applied Biosystems) using an iTaq Universal SYBR Green One-Step Kit (1725151, Bio-Rad) with the following gene-specific primers. For *stat3*, the forward primer is 5′-CCCTGGGACTAACTCTGGCA-3′ and the reverse primer is 5′-AGAGGTCCTGGATTGGCCTC-3′. For *icam-1*, the primers are 5′-CTCAGCATTGAAGTTCACTAT-3′ and 5′-AGATAGACAGTATCGCTCTG-3′. The primers for reference gene *actb1* are 5′-ATGGATGAGGAAATCGCTG-3′ and 5′-ATGCCAACCATCACTCCCTG-3′.

### Compounds

PYR (75 mg/kg; 46706, Sigma Aldrich) or control vehicle was delivered to mice by intraperitoneal injection daily for 15 days. PYR was dissolved in dimethyl sulfoxide (DMSO) to a concentration of 167 mg/ml and then diluted in PBS before injection into mice. For zebrafish studies, 25 µM PYR or vehicle (DMSO) was added to zebrafish embryo water for 3 days prior to assessment of vascular permeability and immunoblotting studies. AQ (A7986, Sigma Aldrich), C188-9 (573128, Sigma Aldrich) and PYR were each dissolved in DMSO and used for *in vitro* studies at the indicated concentrations. For *in vivo* studies, C188-9 was dissolved in DMSO at 208 mg/ml and diluted in 5% dextrose in water (D5W) before injection. Mice received C188-9 (100 mg/kg) or vehicle (DMSO in D5W) by intraperitoneal injection daily for 1 week. For *in vivo* studies, Tyrphostin AG490 (40 mg/kg; T3434, Sigma Aldrich) was dissolved in DMSO at the concentration of 250 mg/ml and diluted in PBS before injection**.** Mice received AG490 or vehicle (DMSO in PBS) by intraperitoneal injection daily for 1 week.

### *In vitro* kinase assay

Human JAK2 protein with active kinase activity (J02-11G-05, Signal Chem) and purified human STAT3 protein from Sf9 cells were subjected to an *in vitro* kinase assay using previously described methodology (Momand et al., 2017). Briefly, 10 µl of JAK2 protein diluted in kinase dilution buffer III (K23-09, Signal Chem) to a final concentration of 0.1 µg/ml was incubated with 3 µg purified STAT3 protein as well as 5 µl ATP (N0440S, New England Biolabs) for 30 min at 30°C. The incubation was terminated by boiling the samples at 95°C for 5 min in 1× Laemmli sample buffer (Bio-Rad) supplemented with 10% β-mercaptoethanol (Bio-Rad). The samples were analyzed by immunoblotting using anti-phosphorylated STAT3 antibody (Y705, Cell Signaling Technology) to validate JAK2-mediated kinase activity upon STAT3 protein at the Tyr705 position.

### Dual-luciferase reporter assay

pGL3-ICAM-1 luciferase reporter (pGL3-ICAM-1-WT) was a generous gift from Dr Jim Hu at the Hospital for Sick Children in Toronto, ON, Canada (Yu et al., 2015). QuikChange Lightning Site-Directed Mutagenesis (Aligent) was performed as described (Alam et al., 2020) to mutate the STAT3 binding site within the ICAM-1 promoter from 5′-TTC-CxG-GAA-3′ to 5′-AGC-CxC-CTG-3′. Constitutively active STAT3 plasmid EF.STAT3C.Ubc.GFP was Addgene plasmid #24983 (http://n2t.net/addgene:24983; RRID: Addgene_24983; deposited by Dr Linzhao Cheng) (Hillion et al., 2008). HUVECs seeded at 1.5×10^6^ cells per well of a collagen-coated six-well plate were transfected with plasmids using the Neon Transfection System (Invitrogen), harvested 48 h post-transfection. Then, Dual-Luciferase Reporter Assays (Promega) were performed, and firefly and Renilla luciferase luminescence was measured using a Synergy Neo 2 Reader (BioTek).

### Generation of stable cell lines

To prepare lentivirus, control shRNA and three different *STAT3* shRNAs cloned in pGIPZ plasmids (University of Minnesota Genomics Center; V2LHS_262105, V3LHS_645974 and V2LHS_88502) were transfected into 293T cells along with their corresponding packaging plasmids. The lentivirus was isolated from cell culture medium 48 h after transfection, concentrated using Lenti-X concentrator (Takara) and used immediately to transduce HUVECs. Stable STAT3 knockdown cells were used for experiments following sorting based on selection of GFP^+^ cells and confirmation of STAT3 protein silencing by immunoblotting.

### GST pull-down assays

The cDNA fragment encoding full-length human STAT3 was subcloned from pLEGFP-WT-STAT3 (Dasgupta et al., 2014) into the mammalian expression vector pEBG (Tanaka et al., 1995) using KpnI and NotI restriction enzyme sites and the following subcloning primer pairs: forward, 5′-GGGGTACCTCGAGCTCAAGCTTCAGGATGG-3′ and reverse, 5′-ATAAGAATGCGGCCGCTCACTTGTAGTCCATGGGGGAGGTA-3′. pLEGFP-WT-STAT3 was Addgene plasmid #71450 (http://n2t.net/addgene:71450; RRID: Addgene_71450; deposited by Dr George Stark) (Dasgupta et al., 2014). pEBG was Addgene plasmid #22227 (http://n2t.net/addgene:22227; RRID: Addgene_22227; deposited by Dr David Baltimore) (Tanaka et al., 1995). FreeStyle™ 293F cells (Thermo Fisher Scientific) grown in FreeStyle™ 293 Expression Medium (12338018, Thermo Fisher Scientific) in an incubator at 37°C with 8% CO_2_ were transfected with pEBG-STAT3 plasmid according to the instructions of the Freestyle 293 Expression System (Thermo Fisher Scientific) and then harvested by centrifugation at 1500 ***g*** for 10 min. Cell pellets were suspended in PBS containing protease inhibitor cocktail (Roche) and lysed on ice using 15 s pulses of sonication repeated seven times with a Sonic Dismembrator, Model 100 (Thermo Fisher Scientific). Lysates in 1% Triton, 5 mM DTT were centrifuged at 12,000 ***g*** for 10 min at 4°C, and Glutathione Sepharose 4B (GE Healthcare) was used to bind the GST fusion proteins from the supernatant. GST protein-coated beads were incubated with pre-cleared HUVEC lysates at 4°C overnight. The bead-protein complexes were then washed five times with pre-chilled PBS, and the proteins were eluted using 10 mM glutathione elution buffer at room temperature. The proteins were boiled for 5 min in the Laemmli sample buffer and then analyzed by immunoblotting.

### Purification of human STAT3 protein

*STAT3* cDNA (human α isoform, residues 1-770) was subcloned from pLEGFP-WT-STAT3 (Dasgupta et al., 2014) into the RGS-6xHis-pcDNA3.1 plasmid (Horn et al., 2014), which was Addgene plasmid #52534 (http://n2t.net/addgene:52534; RRID: Addgene_52534; deposited by Dr Adam Antebi) (Horn et al., 2014). The 6xHis-STAT3α fragment was cloned into the pFastBac™ Dial vector, generously provided by Dr George Aslanidi at The Hormel Institute, University of Minnesota. The plasmid was first transformed into *Escherichia coli* DH10 MultiBac. Single colonies were inoculated into 2 ml antibiotic LB broth containing 50 µg/ml kanamycin, 7 µg/ml gentamycin and 10 µg/ml tetracycline and grown at 37°C overnight. After isolating recombinant Bacmid DNA, we transfected Sf9 insect cells, grew cells in Gibco™ Sf-900™ II SFM medium (10902088, Gibco) in a 27°C incubator, harvested the P0 baculovirus stock, and amplified P1 and P2 baculovirus in a 27°C, 90 rpm shaker. Sf9 cells were infected with P2 baculovirus (multiplicity of infection=10), and cells were harvested by centrifugation at 433 ***g*** for 10 min after incubation for 3 days. The pellet was resuspended in binding buffer [50 mM Tris-HCl (pH 8.0), 500 mM NaCl, 2 mM MgCl_2_, 10% glycerol, 1 mM Tris (2-carboxyethyl) phosphine (T-CEP), 10 mM imidazole] supplemented with complete protease inhibitors (Roche) and then lysed by seven cycles of sonication (Sonic Dismembrator, Model 100, Thermo Fisher Scientific) each consisting of constant pulse for 15 s on ice. The lysate was cleared by centrifugation at 30,000 ***g*** for 30 min at 4°C. The supernatant was subjected to a high-performance HisTrap column (GE Healthcare) using binding buffer (20 mM sodium phosphate, 500 mM NaCl, 20 mM imidazole and 0.5 mM T-CEP) and eluted with an imidazole gradient (0-500 mM). The protein was then concentrated and loaded onto a Superdex 200 size exclusion column equilibrated in 50 mM sodium phosphate, 150 mM NaCl and 0.5 mM T-CEP. Peak fractions were analyzed by SDS-PAGE.

### Statistical analysis

Unpaired, two-tailed Student's *t*-test or one-way ANOVA was used to compare differences between groups as indicated, and values of *P*≤0.05 were considered significant. Before analyzing the data with unpaired Student's *t*-test or one-way ANOVA statistical analysis, we performed normality and lognormality tests followed by Kolmogorov–Smirnov tests using GraphPad Prism8. In all cases, the Kolmogorov–Smirnov tests yielded *P*-values greater than 0.1, so we can conclude that the data conform to a normal (Gaussian) distribution. Bonferroni test was used to correct for multiple comparisons when necessary. Data are expressed as mean±s.e.m. and representative of multiple independent experiments.

## Supplementary Material

Supplementary information
