# Supplementary material for: Suppressing STAT3 activity protects the endothelial barrier from VEGF-mediated vascular permeability
Source: Dis Model Mech. 2021 Nov 11;14(11):dmm049029. doi: 10.1242/dmm.049029 (PMC8592016; doi:10.1242/dmm.049029)
Supplement: Supplementary information [file dmm-14-049029-s1.pdf]

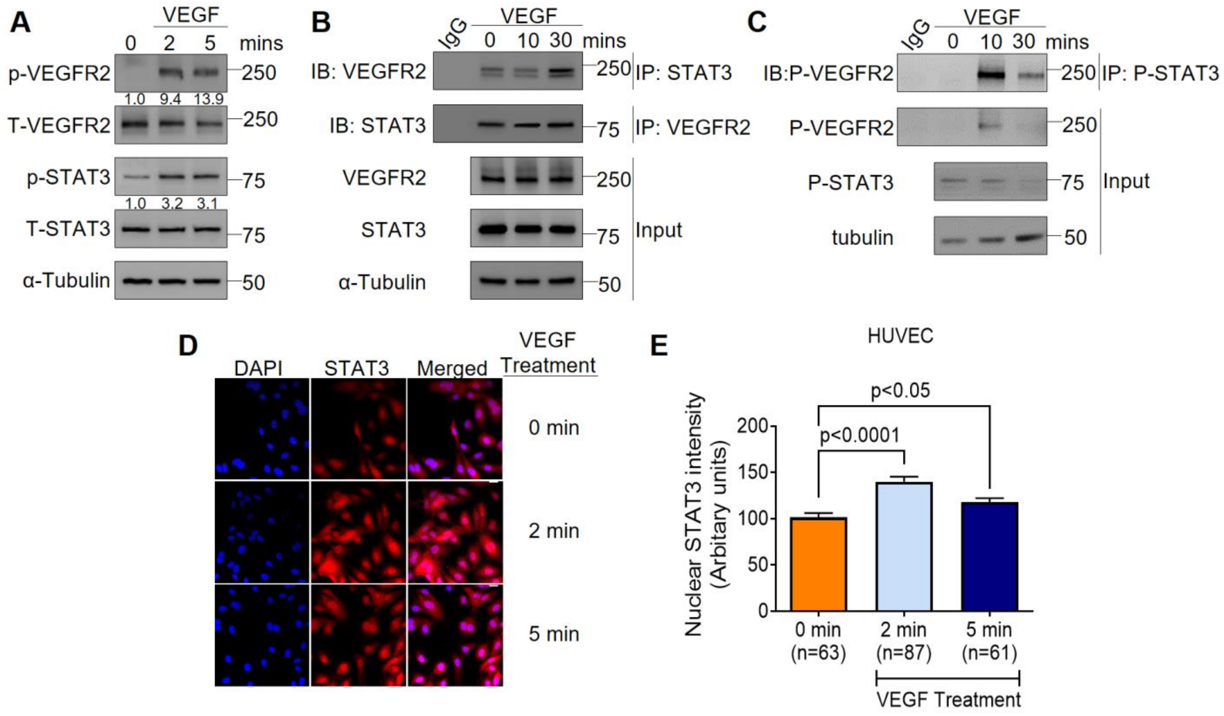

Fig. S1. VEGF/VEGFR-2 induces STAT3 phosphorylation and nuclear localization. (A) Stimulation of HUVEC with 25ng/ml recombinant human VEGF-165 protein for 2 and 5 minutes induces p-VEGFR-2 (Y1175) and p-STAT3 (Y705) via immunoblotting. (B) VEGF (25 ng/ml) stimulation in HUVEC for 10 and 30 minutes promotes co-immunoprecipitation of STAT3 and VEGFR-2. (C) VEGF (25 ng/ml) stimulation in HUVEC for 10 and 30 minutes promotes co-immunoprecipitation of p-STAT3 (Y705) and p-VEGFR-2 (Y1175). (D) VEGF stimulation for 2 minutes and 5 minutes promotes nuclear localization of STAT3. DAPI is blue. Scale bar, 20 µm. (E) Quantification of nuclear immunofluorescence staining intensity. Mean ±SEM, one-way ANOVA followed by Fisher’s LSD test. (A-D) Images are representative of multiple biological replicates.

Disease Models & Mechanisms • Supplementary information

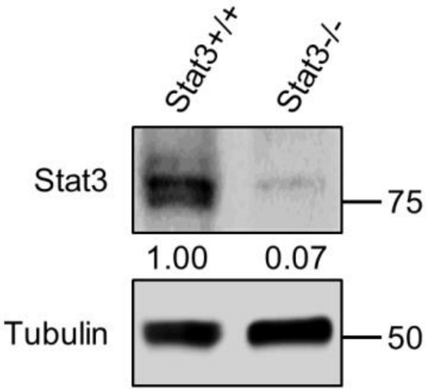

Fig. S2. Validation of Stat3 deficiency in CRISPR-generated Stat3 mutant zebrafish. Stat3 $^{+/-}$ zebrafish were incrossed and embryos were collected. Genomic DNA from a posterior portion of each zebrafish was harvested to verify the genotype. Protein lysates were harvested from the anterior part of the zebrafish embryos by homogenizing with pestles in RIPA buffer after removing yolk sac, and immunoblotting was performed using antibodies against zebrafish Stat3 and Tubulin. Depicted is a representative result of two independent biological replicates.

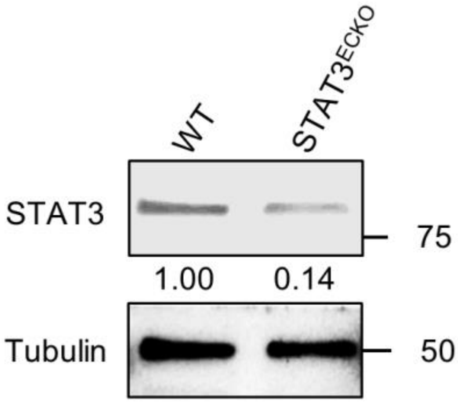

Fig. S3. Validation of endothelial-specific ablation of STAT3 in mice. Endothelial cells pooled from the lung tissues of 4 WT (Tie2 Cre-, STAT3$^{floxed/floxed}$) or 4 STAT3^{ECKO} (Tie2 Cre+, STAT3$^{floxed/floxed}$) mice were incubated with FITC-conjugated CD31 antibody to select for endothelial cells. CD31+ cells were collected through fluorescence-activated cell sorting and lysed in RIPA buffer. Immunoblotting was performed using antibodies against STAT3 and Tubulin.

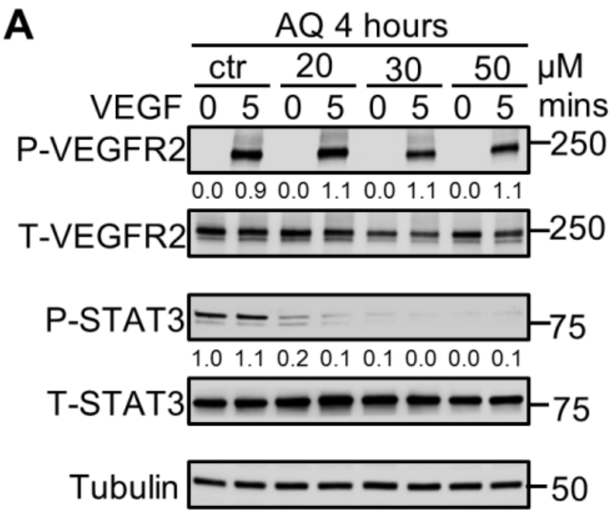

Fig. S4. Assessment of STAT3 inhibition by atovaquone (AQ) and pyrimethamine (PYR) in VEGF-stimulated HUVEC. Serum-starved HUVEC were pretreated with 0, 20, 30, or 50 μM AQ for 4 hours (A) or 0, 10, 20, or 40 μM PYR for 1 hour (B) prior to human VEGF-165 protein (25 ng/ml) stimulation for 0 or 5 minutes. Cells were lysed and then immunoblotted with the indicated primary antibodies. Densitometry was performed and the ratio of phosphorylated protein relative to total protein is reported below the rows of bands. Depicted blots are representative of two independent biological replicates.

Disease Models & Mechanisms • Supplementary information

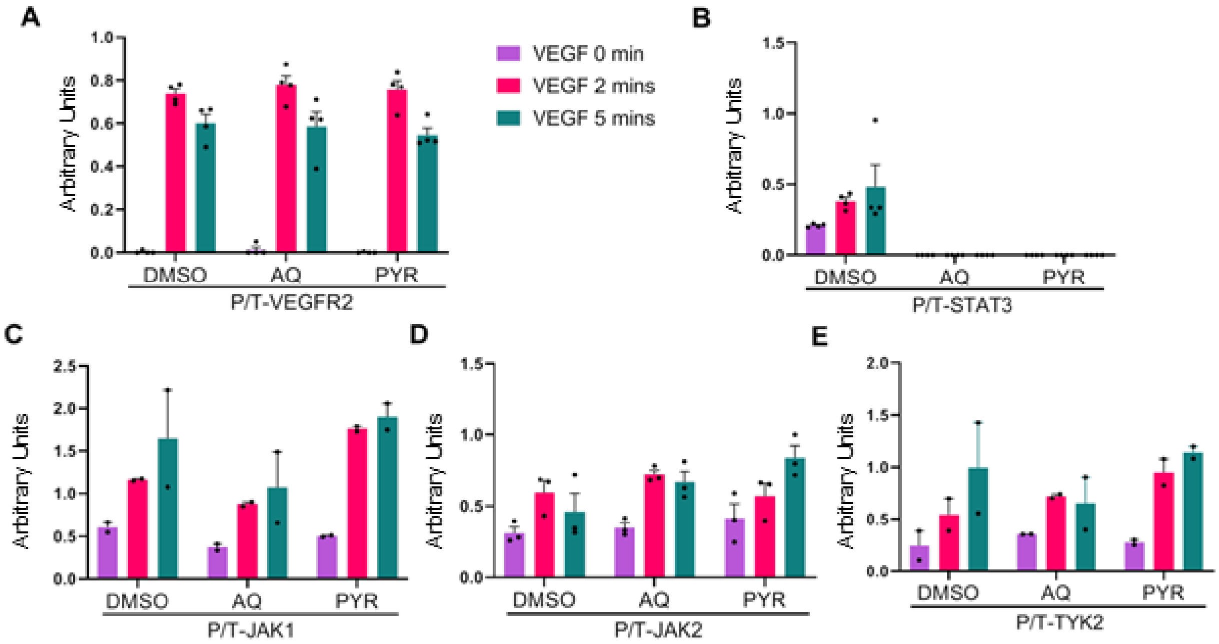

Fig. S5. Densitometry analysis for Figure 3A - Immunoblotting of VEGF-stimulated HUVEC upon pretreatment with STAT3 inhibitors, atovaquone (AQ) and pyrimethamine (PYR). Densitometry was performed on the immunoblotting images by quantitating phosphorylated protein relative to total protein levels as indicated. At least two biological replicates were performed and reported values in the bar graph represent the mean of all the collective biological replicates performed (i.e. each black closed circle represents a densitometry value from one biological replicate).

Disease Models & Mechanisms • Supplementary information

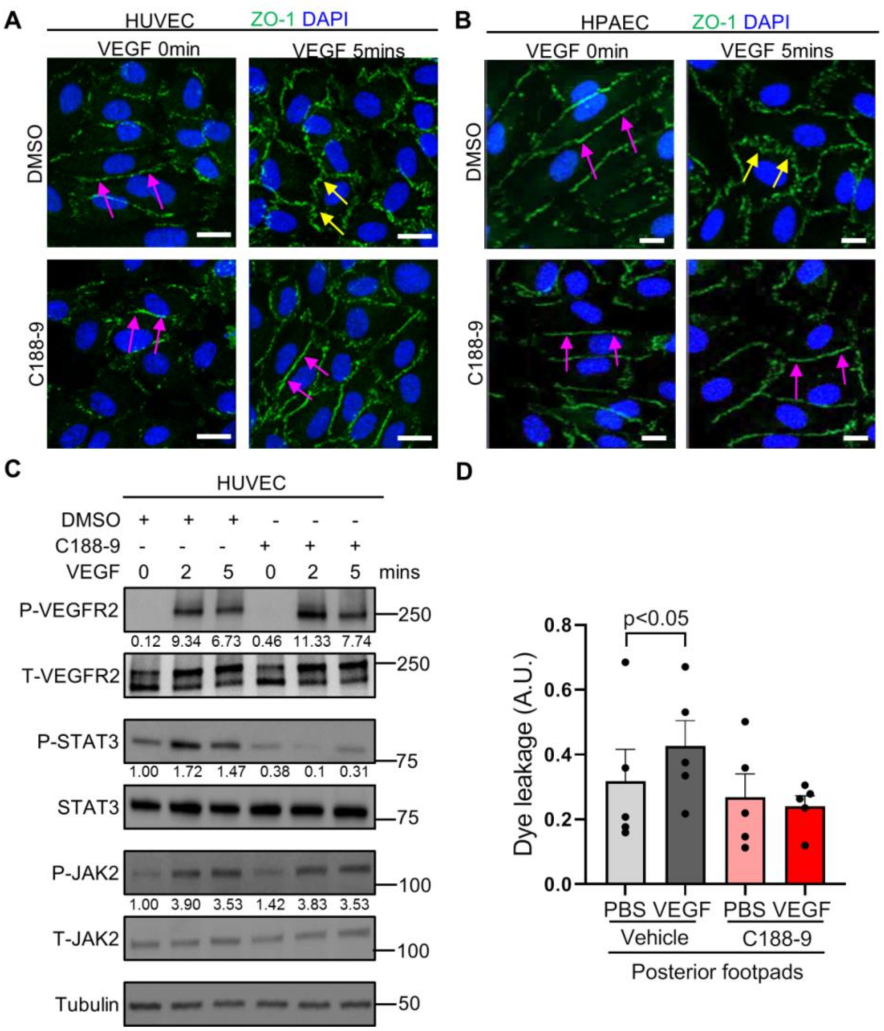

Fig. S6. Pharmacological inhibition of STAT3 stabilizes endothelial barrier integrity following VEGF stimulation in HUVEC and mice. (A) Serum-starved HUVECs were pretreated with 10 μM C188-9 for 5 minutes prior to human VEGF-165 protein (25ng/ml) stimulation for 0, 2 and 5 minutes. VEGF stimulation promotes disorganization of ZO-1 (green) at endothelial cell junctions (yellow arrows). ZO-1 organization is maintained when HUVEC were pretreated with C188-9 (magenta arrows). Nuclei were stained with DAPI (blue). (B) Serum-starved HPAEC were pretreated with 10 μM C188-9 for 5 minutes prior to human VEGF-165 protein (25ng/ml) stimulation for 0 and 5 minutes. After stimulation with VEGF protein for 5 minutes, the structure of tight junction marked with ZO-1 was disrupted (i.e. jagged-like ZO-1 green staining; yellow arrows). ZO-1 organization is maintained when HPAEC were pretreated with C188-9 (magenta arrows). Nuclei were stained with DAPI (blue). (C) Serum-starved HUVEC were pretreated with DMSO (vehicle control) or 10 μM C188-9 for 5 minutes prior to VEGF (25 ng/ml) stimulation for 0, 2 or 5 minutes. Lysates were immunoblotted. The numbers between the bands show the ratio of phosphorylated protein to respective total protein. (A-C) Images are representative of multiple biological replicates. (D) Mice were administered C188-9 or vehicle prior to tail vein injection with 1% Evans blue dye and VEGF (2.5 μg/ml) or PBS vehicle being injected into the root of the footpad. Quantitation of Evans blue leakage in C57BL/6 wildtype mice. n=5 mice per group. Each mouse was injected with PBS on right posterior footpads and VEGF on left posterior footpads. Multiple biological replicates were performed and depicted findings are representative. Mean ±SEM, one-way ANOVA followed by Fisher's LSD test.

Disease Models & Mechanisms • Supplementary information

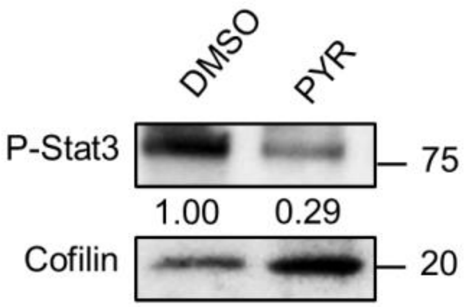

Fig. S7. Pyrimethamine (PYR) inhibits Stat3 protein activity in zebrafish. Zebrafish were exposed to embryo water containing DMSO or 25 μM PYR for 3 days at starting 6 hours post-fertilization. Protein lysates were harvested from 3 days post-fertilization embryos by sonication in RIPA buffer after removing yolk sac and immunoblotting was performed using antibodies against zebrafish P-Stat3 (Y708) and Cofilin (loading control). The depicted image is representative of two biological replicates.

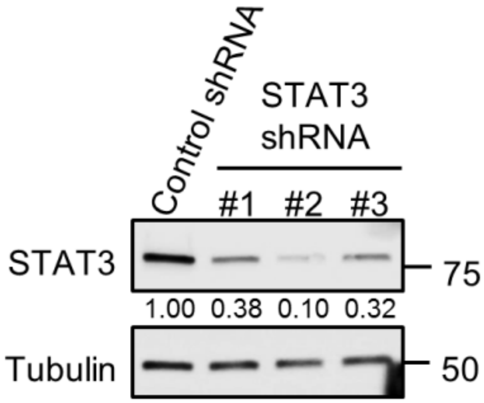

Fig. S8. Validation of STAT3 stable knockdown in HUVEC. Protein lysates were harvested from HUVEC transduced with lentivirus encoding control shRNA or 3 different STAT3-specific shRNAs (i.e. #1, #2, #3) and then immunoblotted with antibodies against STAT3 and Tubulin. STAT3 shRNA #2 shows the best knockdown efficiency. The depicted image is representative of three biological replicates.
